# Antimicrobial Nano-Zinc Oxide Biocomposites for Wound Healing Applications: A Review

**DOI:** 10.3390/pharmaceutics15030970

**Published:** 2023-03-17

**Authors:** Paolo Pino, Francesca Bosco, Chiara Mollea, Barbara Onida

**Affiliations:** Department of Applied Science and Technology, Politecnico di Torino, 10129 Turin, Italy

**Keywords:** wound healing, nanostructured ZnO, biomacromolecules, antimicrobial wound dressings

## Abstract

Chronic wounds are a major concern for global health, affecting millions of individuals worldwide. As their occurrence is correlated with age and age-related comorbidities, their incidence in the population is set to increase in the forthcoming years. This burden is further worsened by the rise of antimicrobial resistance (AMR), which causes wound infections that are increasingly hard to treat with current antibiotics. Antimicrobial bionanocomposites are an emerging class of materials that combine the biocompatibility and tissue-mimicking properties of biomacromolecules with the antimicrobial activity of metal or metal oxide nanoparticles. Among these nanostructured agents, zinc oxide (ZnO) is one of the most promising for its microbicidal effects and its anti-inflammatory properties, and as a source of essential zinc ions. This review analyses the most recent developments in the field of nano-ZnO–bionanocomposite (nZnO-BNC) materials—mainly in the form of films, but also hydrogel or electrospun bandages—from the different preparation techniques to their properties and antibacterial and wound-healing performances. The effect of nanostructured ZnO on the mechanical, water and gas barrier, swelling, optical, thermal, water affinity, and drug-release properties are examined and linked to the preparation methods. Antimicrobial assays over a wide range of bacterial strains are extensively surveyed, and wound-healing studies are finally considered to provide a comprehensive assessment framework. While early results are promising, a systematic and standardised testing procedure for the comparison of antibacterial properties is still lacking, partly because of a not-yet fully understood antimicrobial mechanism. This work, therefore, allowed, on one hand, the determination of the best strategies for the design, engineering, and application of n-ZnO-BNC, and, on the other hand, the identification of the current challenges and opportunities for future research.

## 1. Introduction

Wounds are a major global healthcare issue that have been defined as a “silent epidemic” [1,2] for their prevalence and profound effects on global health and patients’ lifestyles and psychological wellbeing. Overall, it has been estimated that chronic wounds alone affect 1–2% of the population in developed countries [1]. This problem is set to worsen as the incidence of chronic wounds is correlated with age and other age-related co-morbidities, such as diabetes. In European Union countries, wounds have an annual incidence of 4 million people [3]. In a study carried out across several countries, 65% of which were European, chronic wound prevalence was found to be 1.67 per 1000 individuals [4]. Chronic wounds are also deadly: a study by Eaglstein and co-workers [5] reported that chronic wounds, such as diabetic ischemic ulcers, diabetic foot ulcers, and lower extremities chronic wounds, have mortality rates of 52%, 49%, and 28% respectively, comparable with those of several types of cancer.

The economic implications are also relevant. In developed countries, chronic wound care costs account for 1–6% of total healthcare expenditures [6]. 

Burns are another type of concerning wound. According to the World Health Organization, 180,000 people die every year because of burn injuries, most frequently in low-income countries [7].

In the United States alone, the annual cost of acute and chronic wound care ranged from USD 28.1 billion to USD 96.8 billion in 2018 [8]. This also implies massive and indirect economic burdens correlated with decreased productivity, early retirement, and wage losses [9]. 

The severity and mortality of wounds are also expected to increase due to their exposure to antimicrobial-resistant pathogens. A study by Sen [8] reported how wound infections already cause 75% of post-operative deaths. As far as burns are concerned, while the mortality rate has decreased over the years due to improvements in medical techniques (such as fluid resuscitation, nutritional support, early excision of the wound eschar, and skin grafting) [10], infection and sepsis are still common and often fatal: 51% to 75% of patients who experience a major burn die [10,11]. This fact is, in part, attributed to impaired immune system functioning [12]. With the rise of antimicrobial resistance (AMR), treating wound infections is becoming increasingly harder [13]. It has already been discovered that 90% of the *S. aureus* strains isolated from wounds worldwide in nosocomial environments are resistant to penicillin [14]. *S. aureus*, *E. coli*, and *P. aeruginosa* are the most common pathogens isolated from infected wounds, showing very high rates of single- or multi-drug resistance [15,16].

This calls for advanced wound dressing solutions. In time, the functions and features of wound dressings have evolved from passive protection of the injured site from the external environment to active involvement in the healing process, mediated by the ability of these advanced materials to interact, both physically and biochemically, with the surrounding tissues and environment, for instance, by triggering cellular proliferation pathways or by eradicating bacterial infections [17,18]. In these regards, bionanocomposites (BNCs) are gaining increasing attention owing to their promising biomedical properties. BNCs are composite materials whose matrix is constituted by biomacromolecules, i.e., nature-derived macromolecules with bioactive features and the ability to form films, hydrogels, or fibres. In fact, traditional generic wound dressings, such as sterile gauzes, have been replaced over time by new dressings with enhanced ability to promote healing and that are targeted for specific wound types [19]. For instance, foams and hydrogels have been developed for highly exudating wounds, while hydrocolloids are recommended to support wound debridement. Films are preferred when flexibility, transparency and gas permeability are important, such as in the case of burns [20,21]. Biomacromolecules are very well suited for the fabrication of these devices, and intense research activity is undergoing on the development of films, hydrogels, and fibrous membranes. In many cases, these molecules are also very abundant and easy to source, which makes them a competitive alternative to petroleum-derived compounds, also from an economic standpoint. The dispersed phase of BNCs is instead represented by nanomaterials, materials which have at least one of their dimensions in the nanoscale, i.e., below 100 nm. Under this condition, new properties are, disclosed enabling unprecedented applications. One of these properties is the much higher surface-to-volume ratio and specific surface area compared with that of bulk materials. As materials interact with the outer environment through their surface, a larger surface means a larger number of interactions and a larger surface energy, and, therefore, greater reactivity and efficacy in several applications [22]. The specific surface area can also be largely increased in the presence of pores, which can then be filled with other substances, such as drugs or antibiotics for targeted and controlled delivery. Another important property is the reduced size, which allows nanoparticles (NPs) to diffuse more effectively in the extracellular environment and to get in contact with—or, in some cases, to penetrate—the surrounding cells [22].

During the last few years, many nanomaterials have been obtained, designed, and studied for wound-healing and antimicrobial applications. For the latter, a major parting line can be drawn between carbon-based and metal-based nanomaterials. The first group includes carbon nanomaterials, such as carbon quantum dots, carbon nanotubes, graphene, and fullerenes, which have all shown antimicrobial and skin regeneration-promotion effects [22,23]. Lipid and polymer NPs can also be included in this category, although they are mostly applied as carriers in drug delivery. The second group includes metal NPs—namely silver and gold NPs—and metal oxide NPs, such as iron, copper, titanium, and zinc oxide NPs. These NPs have been proven to be effective in eradicating multi-drug-resistant species and in promoting wound healing and re-epithelialization [22,23]. Nanoparticles are frequently embedded into BNC hydrogels, foams, and films as a strategy to leverage their wound-healing and antimicrobial properties [24], but they can also be used to coat suture materials [25] or be applied in the form of gels and stabilised dispersions [26] or injectable aqueous suspensions [27]. Among these, nanostructured zinc oxide (nZnO) has shown anticancer [28,29], antimicrobial, anti-inflammatory [30], and anti-diabetic properties [31], as well as applications in bioimaging [32], even though some biocompatibility concerns have been recently brought to the attention of the scientific community [33]. Contrary to other antimicrobial materials, such as gold, silver, or titanium dioxide NPs, nZnO shows lower toxicity [23] and acts as a supply of zinc, which is an essential element for the human body owing to its role in the proper functioning of metabolism, immune system, and wound healing processes [34,35,36]. 

**Figure 1:**
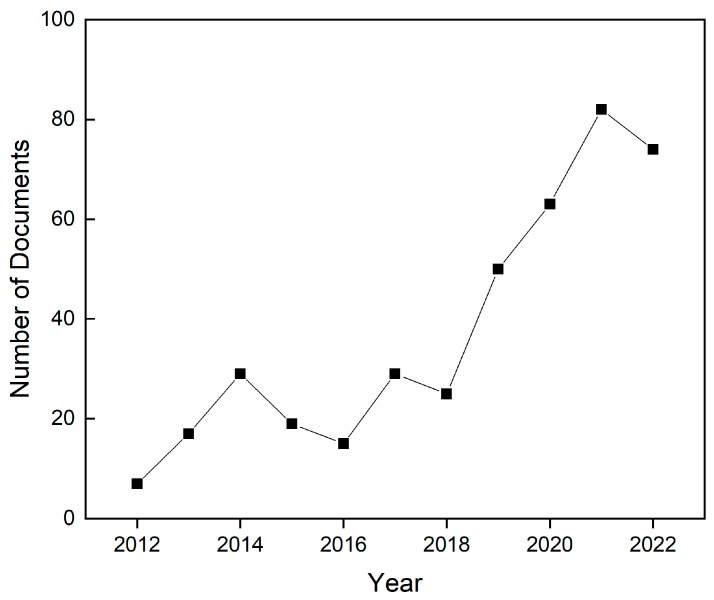
Number of published scientific articles, reviews, and book chapters on the topic of antimicrobial zinc oxide composite films and membranes for wound healing from 2012 to 2022. Source: Scopus.

For all these reasons, despite some concerns existing around the toxicity of nanoparticles for human health [37], nZnO has great potential to positively impact wound care. Many nanocomposite films, patches, membranes, and bandages have been designed using nZnO as a filler, and the number of research studies addressing the antimicrobial uses of these materials has been rising steadily over the past 10 years, as shown in Figure 1. 

The present review considers the work conducted since 2012 to gain deep insights into the latest advancements in the development of antimicrobial nZnO-BNC films, with the goal of identifying common traits and emerging opportunities, as well as gaps and open questions in research. Films have been considered with particular attention as they do not experience the drawbacks of hydrocolloids and some hydrogels (wound maceration and bad smell) or foams (painful removal and excessive drying of the wound bed) [23]. Previous works have addressed this topic with a particular focus on the most diffused natural polymers used as matrixes in the fabrication of antimicrobial nZnO-BNCs, such as chitosan, alginate, and cellulose [38]. However, other materials are being increasingly used, and more sophisticated combinations of polymers and fillers are being investigated. Similar efforts in this direction have been made by Zahran et al. [39] for nanocomposites containing metal NPs, mostly silver NPs. In this study, biomacromolecules, such as cellulose, starch, chitin, chitosan, dextran, gelatin, alginate, pectin, guar gum, rubber, and fibrin, were considered.

Special attention here is given to the relationships existing between the matrix and the nZnO filler as key factors in the successful design of nanocomposite films, and to their influence on the resulting physico-chemical and antimicrobial properties. 

## 2. Biomacromolecules for Wound Dressings

### 2.1. Overview

As mentioned in the introduction, biomacromolecules are particularly promising for the fabrication of novel, more effective antimicrobial wound dressings. Contrary to the petroleum-derived synthetic polymers, traditionally utilised, biomacromolecules are more biocompatible and conductive towards re-epithelialization, and can be easily and sustainably processed into films and hydrogels [40]. When these properties are combined with the antibacterial and wound-healing action of nZnO, new biocomposite materials emerge. Biocomposites provide better conditions for injured tissues to re-grow while preventing infections, decreasing healing times, and improving patients’ health. Many biomacromolecules, alone or in a blend, have been used for nZnO-BNC film and dressings obtaining different properties and peculiarities. An overview has been reported in Table 1.

Among these, some have attracted more attention in the scientific communities, which can be classified into two major categories: polysaccharides and proteins.

### 2.2. Polysaccharide-Based Polymers 

Chitin is a natural polycationic polymer of N-acetylglucosamine. It is typically obtained from the cuticles of crustaceans, insects, and cell walls of fungi [88,89]. Chitin deacetylation produces chitosan, a renowned antimicrobial material [90] that is also active against some resistant strains, such as the methicillin-resistant *Staphylococcus aureus* (MRSA) [91]. Chitosan is biocompatible, non-toxic, haemostatic, and, thus, frequently used in biomedicine, also for wound healing [92,93,94,95] and the delivery of drugs and biomolecules [96]. Owing to these properties and due to its good processability, it is often mixed with other polymers, such as poly(ε-caprolactone) [97,98], polyurethane [99], or poly(vinyl)alcohol [100] to produce advanced wound dressing films, hydrogels, and membranes. It also lends itself very well for use in new techniques, such as electrospinning [101,102]. 

Cellulose is a polymer of β-bonded D-glucose units that can be obtained from plants or synthesised by bacteria [103,104]. It is non-toxic, biocompatible, and biodegradable. It is water-insoluble and possesses good thermal stability. It is gaining increasing attention, particularly in its nanostructured forms [105,106,107,108], and extensive research exists exploring drug loading and combination with nanomaterials [109,110,111]. Gopi and Zhong offered a wide review of its uses and applications in the medical field, including wound dressing and drug delivery [104,112]. Here, bacterial cellulose emerged as particularly promising for the fabrication of films and fibrous mats for wound therapy [113,114]. Several cellulose derivatives can also be obtained by modifying cellulose molecules with different functional moieties, such as carboxyl [115] and allyl [116] groups. This allows the manipulation of key properties, such as hydrophilicity, or enabling its conjugation with drugs or other medical compounds.

Hyaluronic acid, a glycosaminoglycan, is a component of the extracellular matrix. It is typically extracted from animal tissues or produced by the fermentation of some *Streptococcus* strains [117,118].

Its biocompatibility and hydrophilicity are valuable for the development of biomedical materials, especially hydrogels [119]. Recently, Graça and coworkers [120] offered a review of its applications in wound dressings, underlying its highly beneficial role in wound repair and cell signalling [121,122]. Another review by Ucm focused on the synthesis of hyaluronic acid by bacteria and its applications—including wound healing [123].

Alginate is a linear co-polymer constituting two monomeric units of D-mannuronic acid and L-guluronic acid. It shares similar properties [124] with hyaluronic acid, namely its high biocompatibility and hydrophilicity. Commercial alginate is isolated from brown seaweeds, and its bacterial production has recently gained interest [125]. Its high water-absorption capacity has been known for a long time; thus, this biomacromolecule has been extensively used in the production of wound dressings for high-exudate wounds. Alginate stimulates macrophages’ activation, supporting the healing process [19]. The abundance of hydroxyl and carboxyl groups in its molecule provides this polymer with high reactivity, which is also useful in the design of encapsulation and drug-delivery systems [124]. Its biocompatibility and printability meet the need for biomaterials for tissue engineering and modern wound dressings [126,127,128].

Starch is an abundant, plant-derived polysaccharide composed of α-bonded glucose units. It is endowed with biodegradability, non-toxicity, and film-forming ability. It can be processed with simple extrusion or solvent casting methods to obtain films [129], as well as with electrospinning [130]. 

β-glucans are typically found in the cell walls of fungi, yeasts, algae, and plants. They are a heterogeneous group of glucose polymers with a common structure comprising a main chain of β-(1,3) and/or β-(1,4)-glucopyranosyl units, along with side chains with various branches and lengths.

They are known for their immunostimulatory properties [131] and they have been shown to stimulate collagen deposition, fibroblasts and keratinocytes migration, and overall reepithelialisation [132,133,134]. Therefore, these polysaccharides have been recently researched to produce advanced dressings, such as wet gels and nanofibers for hard-to-treat and chronic diabetic wounds, as well as for antimicrobial films [74,135,136].

### 2.3. Protein-Based Polymers

Protein-based biopolymers, such as collagen and gelatin [83,137], are also very popular. Collagen is the main constituent protein of the extracellular matrix and is extracted from animal skin and hides. It is a source of many bioactive peptides. It favours tissue regeneration, has good moisture-retention properties, and has been used for the controlled release of bioactive molecules [40]. Several collagen films have been described in the literature for wound healing and tissue engineering [138,139,140]. Gelatin is a heterogeneous mixture of peptides derived from collagen. It has good film-forming ability, transparency, and ease of combination with functional and reinforcing additives or other biopolymers [141,142]. It has also been used to fabricate scaffolds [143,144], aerogels [145], and antimicrobial nanofibrous composites [146].

Keratin is a fibrous protein that is usually extracted from feathers, nails, horns, or wool [147]. This has raised concerns around the extraction methods and the environmental issues related to the accumulation of waste keratin-containing biomasses [148,149]. It has been extensively researched for the fabrication of films, hydrogels, and fibres, with uses in wound healing, drug delivery, and tissue engineering owing to its biocompatibility, biodegradability, and self-assembly properties [150,151].

Whey proteins are biocompatible and biodegradable byproducts of the dairy industry and are mostly used as food additives and supplements. Their excellent film-forming ability and gelation properties have been extensively investigated and used for the formulation of edible films and particles for the delivery of nutraceuticals. The combination of whey protein isolate (WPI) with nZnO has been described in a previous study [152].

## 3. Nanostructured Zinc Oxide (nZnO)

As mentioned in the Introduction section, nZnO has shown many promising biomedical properties, such as anticancer, antibacterial, antifungal, antidiabetic, and anti-inflammatory effects, and has been used for imaging and drug delivery. Owing to the extensive literature and review works available on nZnO [36,153,154], this section aims to briefly recall its main characteristics, with special attention towards its antimicrobial properties.

### 3.1. Preparation Methods

Nano-ZnO can be produced through a wide variety of techniques. such as thermal evaporation [155], sol-gel [156,157], mechanochemical [158], sonochemical [159], and microfluidics methods [160]. Microwave-assisted syntheses [161,162,163,164] are also gaining increasing attention due to the shorter reaction times and better size and morphology control with respect to conventional chemical routes. Wet chemical precipitation is, however, the most common method for nZnO in nanocomposite preparation, as it is often cheaper, simpler, and yields higher amounts of NPs. It involves the reaction of a zinc precursor—usually zinc nitrate [58,165], zinc sulphate [166], zinc chloride [167], or zinc acetate [66,157]—with a base solution—such as KOH or NaOH. The reaction takes place under very mild conditions, typically at ambient pressure and constant temperatures up to 80 °C [168]. The precipitate is subsequently collected, washed, and finally dried or calcinated [169].

Recently, a growing trend in nanomaterials and nZnO synthesis has been the use of green compounds, such as plant extracts [170,171,172,173,174,175,176,177,178,179,180], to replace NaOH and KOH as reducing and capping agents. The extracts that can also functionalise NPs with phytochemicals possessing anti-inflammatory, antioxidant, or antimicrobial properties, further mitigating potential toxicity issues.

**Figure 2:**
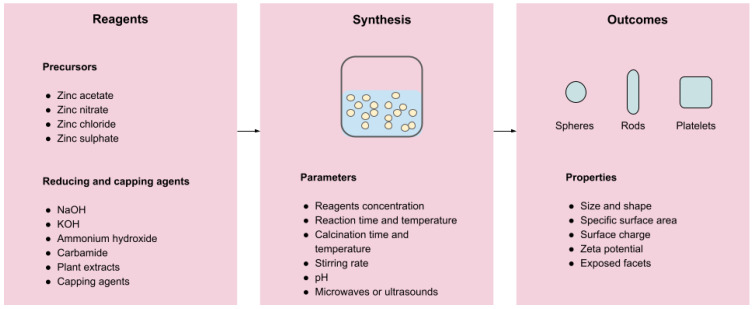
nZnO design through the selection of reagents and synthesis parameters.

Ultimately, the selection of reagents and synthesis parameters shapes the final product in multiple ways. Parameters, such as the reaction or calcination temperatures, pH, or stirring rate, have all been proven to influence the final size and shape of the nanoparticles, as well as their surface charge, crystallite size, or porosity [156,181]. Figure 2 below summarises how and where these factors are involved in the preparation process.

### 3.2. Antimicrobial Mechanisms

**Figure 3:**
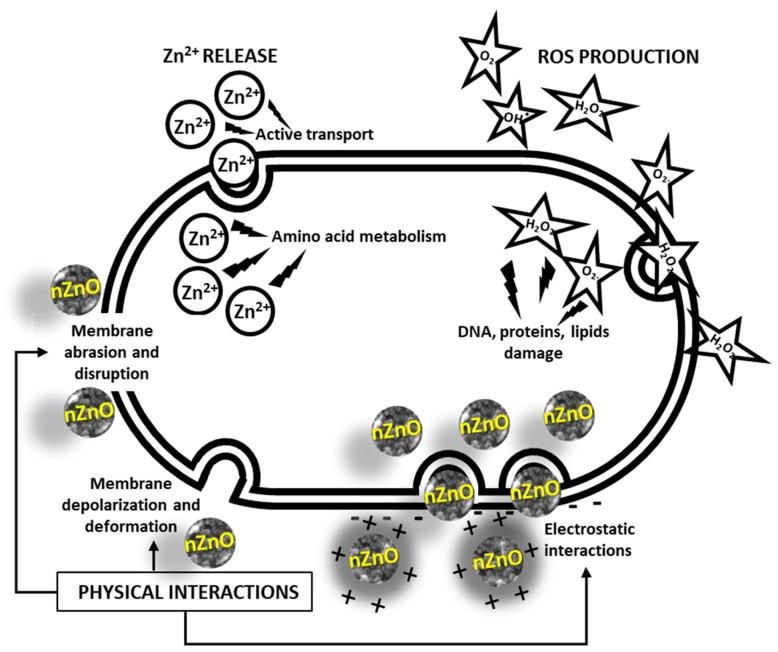
Antibacterial mechanisms of nZnO. Modified from [182].

Nano-ZnO fulfils its antimicrobial action through three main mechanisms:

- Release of Zn^2+^ ions. These ions bind to the thiol groups of bacterial respiratory enzymes, compromising their activity [36]. They have also been found to inhibit active transport across the plasma membrane and amino acid metabolism [153] in bacteria.
- Production of Reactive Oxygen Species (ROS). Zinc oxide is a direct, 3.3 eV-bandgap semiconductor. Therefore, electron–hole pairs can be created across its conduction and valence bands upon exposure to UV radiation. In the presence of water and oxygen molecules, these charge carriers subsequently trigger redox reactions at the surface of the material, leading to the production of ROS, such as hydroxyl radicals OH∙, hydrogen peroxide H_2_O_2_, and superoxide anion O_2_^−^. The high reactivity of these species is known to be responsible for the fatal oxidative damage and disruption of DNA, proteins, and lipids, ultimately leading to cell death [153,154].
- Physical interactions. ZnO NPs accumulate in the proximity of bacterial cells. The contact is promoted by electrostatic interactions. In fact, bacterial cells are negatively charged, while ZnO NPs typically possess a positive charge in aqueous suspensions. In turn, this causes membrane depolarization and deformation, as well as abrasion caused by nZnO’s sharp surface edges, ultimately leading to cell death [153]. A graphical representation of these three mechanisms is offered in Figure 3.

As a rule of thumb that emerged over years of experimental studies on the antimicrobial activity of zinc oxide, nZnO tends to be more effective against Gram-positive species than Gram-negative ones [183,184]. This is attributed to the structural differences in cell walls that characterise the two types of bacteria. Gram-positive bacteria have a thick layer made of peptidoglycans surrounding the cellular membrane. In Gram-negative bacteria, the peptidoglycan layer is thinner, but it is, in turn, surrounded by an additional phospholipid outer membrane. The outer membrane is often considered to be responsible for the highest resistance of Gram-negative bacteria to nZnO, as it is hypothesised to act as a shield against Zn^2+^ and ROS. It has also been found that small concentrations of ZnO can actually enhance the growth of Gram-negative bacteria [153]. This is a crucial aspect to be considered; on one hand, it restricts the potential use cases of this nanomaterial; on the other hand, it offers a clear direction for improvement.

It is also important to highlight that the features of nZnO determine the contribution of each of these mechanisms to the final antimicrobial action. In other words, nZnO—and nanomaterials more in general—can be designed to maximise its efficacy.

In fact, factors such as the selection of reagents or the stirring rate [156] ensure a good degree of control of the final characteristics of the NPs, leading to a wide variety of sizes and shapes, such as nano-spheres [157], nano-rods [178], nano-platelets [185], and nano-flowers [179]. These morphological features, together with the specific surface area (SSA) and surface electric charge, have all been found to contribute to nZnO antimicrobial activity, as reviewed by several authors [38,153,175,180]. For instance, a smaller size and a spherical shape have been found to increase NPs’ solubility, thus boosting the release of Zn^2+^ ions. Similarly, the type of facets exposed—e.g., (100) versus (111)—have different antimicrobial activities and can thus be considered when developing new types of nZnO [153]. 

Here, it is crucial to highlight that toxic effects might also arise against eukaryotic cells. As for fungi, similar mechanisms to those described above for bacteria are considered responsible for the antifungal activity of nano-ZnO [45,186]. Several studies have examined the cytotoxicity of nZnO to human cells [33,158,187]. They attributed the toxic effects to the upregulation of the p53 protein pathway in the presence of nZnO, with the subsequent induction of cellular apoptosis. This is considered a response to the oxidative stress exerted on DNA by ROS, which have been described by several authors as the main toxicity driver in mammalian cells, together with Zn^2+^ release.

Now that the main features of nZnO and biomacromolecules have been recalled in relation to their antimicrobial and wound-healing applications, it is time to explore their synergies when they are combined to form BNCs.

## 4. Biomacromolecule—nZnO Composites

### 4.1. Preparation Methods

Several preparation processes exist for nZnO-BNC films or hydrogels. Zahran et al. [39] offered a useful overview of the preparation methods for polymer–nanoparticle nanocomposites. Following that classification, two options are found to be most common for nZnO-BNCs, namely in situ and ex situ methods.

In situ methods consist of mixing a suspension of nZnO with a monomer solution or polymeric chain suspension [188]. After sufficient homogenisation of the two components, the BNCs can be obtained by entangling or crosslinking the polymers, or by polymerizing, reticulating, or gelling the monomers. This can be achieved through the addition of a polymerization initiator or crosslinking agent [75], or by solvent evaporation. In this latter case, solvent casting [48,56,65,68,71,84,99,189,190] is the most common technique to produce composite films. Freeze-drying [41,191,192] is more used for hydrogel membranes and patches, while electrospinning [69,193,194], spin-coating, or electrophoretic deposition [195] can be applied to obtain fibrous mats.

In situ methods can be used with low-molecular-weight, well-soluble polymers and facilitate a good dispersion of the filler. They have been successfully used to produce, among others, gelatin/hyaluronic acid/chitosan [83], cellulose [196], starch [62], and keratin [79] composite hydrogels containing nZnO, as well as WPI films [152]. Moreover, they are the most common methods for obtaining polyurethane–ZnO composite films, membranes, and coatings, as demonstrated in the dedicated review work conducted by Rahman [197].

Ex situ methods consist of immersing an already-formed matrix into a nZnO suspension under agitation in order for the nZnO to migrate inside the matrix and to finally interact with it, either physically or chemically. These methods are relatively simple, but are limited to highly porous matrixes with good affinity for the suspension medium, and are capable of significant swelling in that environment. Indeed, poor penetration of NPs in the core region of the matrix is a typical issue. Ex situ methods have been applied to craft, for instance, keratin/chitosan [78] hydrogels, or alginate bandages [41].

**Figure 4:**
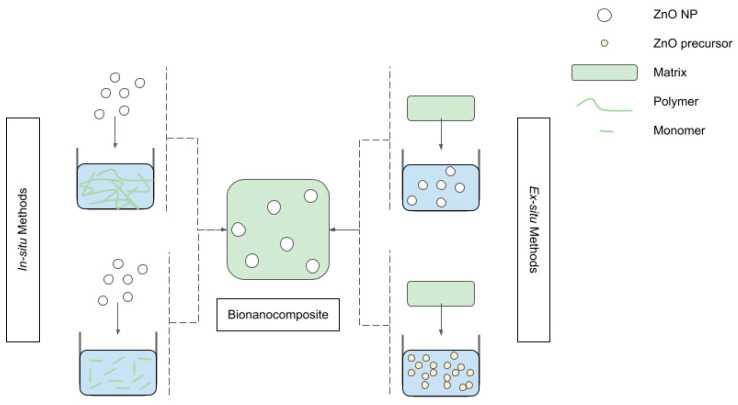
Schematic representation of BNCs’ preparation techniques.

Several variations of both the above-described methods have been adopted by researchers. In some cases [75,198], for in situ methods, the direct addition of pre-synthesised nZnO is replaced by the addition of a zinc oxide precursor. This leads to a solution of monomers and zinc ions, which is then brought to basic pH to favour the simultaneous formation of nZnO and polymers. Similarly, in some ex situ routes, the preformed matrix is immersed into a zinc oxide precursor solution [58,64]. This allows Zn^2+^ ions to diffuse into the polymeric network before the formation of ZnO. Figure 4 summarises the main approaches to BNCs’ fabrication.

Once together, the interaction between nZnO and the matrix is mostly regulated by hydrogen bonds and by van der Waals interactions [65,71,73,75,80,189,192,199]. This plays a significant role in shaping the final nanocomposite and its properties. For instance, a decrease in matrix crystallinity has been reported in several BNCs made from chitosan or cellulose [50,53,100,158,189,191,200,201]. This has been ascribed to the disruption of biomacromolecular order caused by nZnO. In fact, these biomacromolecules are rich in amino and hydroxyl groups, which, upon interaction with (or shielding by) nZnO, are no longer available to mediate the intermolecular interactions governing the spatial organisation and subsequent crystallization of the macromolecular chains.

It is interesting to note how an increase in crystallinity has been reported for some synthetic poly(vinyl alcohol)-based nanocomposites [52,192,202], as well as in composites of non-ionic polymers, such as cellulose acetate, poly(butylene-succinate), and poly(lactic acid) [69,203,204]. In these cases, given the minor contribution of electrostatic phenomena, the stabilizing effect of nanophase might prevail, facilitating molecular organisation and triggering heterogeneous nucleation.

Conversely, when nZnO synthesis is carried out within the BNCs’ preparation process starting from a precursor, either in situ or ex situ, the resulting nanofiller is affected in several ways. The existence of an already-formed matrix or the presence of monomers or macromolecules in the solvent can interfere with nZnO formation by acting as capping agents that constrain the growth of the NPs and regulate their final shape [75,205].

### 4.2. Mechanical and Functional Properties

Following the above description of preparation methods, we shall now dive into how the inclusion of nZnO inside a biomolecular matrix translates into new or modified properties.

#### 4.2.1. Mechanical Properties

Mechanical properties are critical for antimicrobial BNCs for wound healing. Inadequate mechanical properties can result in low compliance with the skin and in the inability to stretch or bend according to patients’ movements, as well as in poor protection from scratches and punctures. They can also limit the suitable production technologies and drive an increase in the relative costs.

In general, the addition of nZnO typically has a concentration-dependent reinforcing effect translating into an increase in tensile strength and elastic modulus, accompanied by a decrease in elongation at break. A 176% enhancement of tensile strength was reported by Azizi [52] after reinforcing a poly(vinyl alcohol)/chitosan blend film with nZnO and cellulose nanocrystals. The polyurethane/chitosan film made by Indumathi [99] achieved a 56% improvement in tensile strength after a 5% *w*/*w* nZnO addition. Similarly, a 5% *w*/*w* nZnO loading allowed increases in the tensile strength and the Young’s modulus of a bacterial cellulose composite film of 42% and 30%, respectively, according to Jebel et al. [53]. Arfat and co-workers achieved a 36% tensile strength increase by adding 3% *w*/*w* nZnO to a fish protein isolate/fish skin gelatin composite film [81]. Similar results have been obtained by other researchers as well [65,69,80,99,158,190,191,192,203,206]. The reasons underlying this reinforcement are ascribed to the ability of nZnO to limit the movement of polymeric chains by means of physical, chemical, and mechanical interactions. This effect is further enhanced by a high degree of dispersion of the filler and by a high surface area, which increases by decreasing the size of the NPs.

On the other hand, a worsening of mechanical properties has been reported in some cases, occurring above the initial concentration-dependent improvement, at the highest nZnO contents [71,100,189,207]. In fact, after a critical concentration threshold, the agglomeration of nZnO into larger aggregates is triggered, thereby causing a worsened ability to transmit loads across the composite microstructure. The use of a capping agent wrapped around nZnO has been shown to mitigate these side effects by preventing agglomeration and favouring dispersion [56].

Aside from the role of the nZnO content, a weak matrix–filler interaction causes a decrease in the mechanical properties, where the resulting nanocomposite shows higher elongation at the break and decreased tensile strength and elastic modulus, as reported by Kanmani et al. [68] for carrageenan, carboxymethyl cellulose, and agar-based composites obtained in situ via solvent casting. Similar results were obtained by other researchers [66,73,204]. A summary of these findings is offered in Table 2.

#### 4.2.2. Surface Roughness and Wettability

Many of the studies examined in this work found that the incorporation of nZnO into the matrix usually leads to an increase in the surface roughness due to the presence of the nanomaterial in the upper layers of the film and at the interface with the external environment [80,99]. This has an important impact on another important property, that is, wettability.

Wettability is defined as the affinity of a given surface for a liquid, typically water. The traditional measure of wettability is the water contact angle (WCA). There is wide agreement in the community that the addition of nZnO to biopolymeric materials causes an increase in the WCA, meaning an increase in hydrophobicity [66,68,71,99,190,192,208,209]. One of the causes is, indeed, the increase in the surface roughness, which opposes capillary forces to the penetration of water droplets into the material. Another cause is the lower water affinity of nZnO compared with biomacromolecules. A third reason is, in some cases [192], the enhanced crystallinity of the surrounding matrix: more tightly packed crystalline phases are more difficult to be penetrated by water. A high water affinity—and thus a low WCA—is important for wound dressings as it ensures good adhesion to the underlying tissue. In many cases, it goes in parallel with the ability of the dressing to absorb wound exudate. Given that nZnO increases the WCA, it could be argued that its effect is, in fact, detrimental on this important feature of a good dressing. However, the emergence of a hydrophobic behaviour upon the addition of nZnO, i.e., a WCA greater than 90°, is extremely rare. In most cases, hydrophilicity is preserved, as shown in Table 3.

#### 4.2.3. Porosity and Swelling

Porosity is also influenced by the presence of nZnO, and both increases [51,55,75,78] and decreases [41,53,73] in porosity were reported by authors.

A high porosity may be beneficial for the swelling ratio (SR)—defined as the percentage increase in the weight of a specimen after immersion in a liquid medium with respect to the dry weight. SR has been reported to increase in composite films and hydrogels after the addition of nZnO [51,55,58,75,79,192]. In addition to a higher porosity, this was also attributed to the electrostatic charge on the surface of nZnO, which causes ionic osmotic pressure build-up inside the matrix, thereby inducing higher water uptake to counterbalance it. However, high concentrations of nanofiller might too-strongly reduce the intermolecular movements at the basis of the swelling phenomenon—the so-called knot-tying function—thereby reducing the swelling ratio in some cases [41,43,61,64,73,196,211,212]. Another reason for this decrease in the swelling ratio of nanocomposites can be the overall lower water affinity of the system due to the diminished net electrostatic charge of the matrix caused by the shielding effect imparted by nZnO.

#### 4.2.4. Gas Barrier Properties

Barrier properties to oxygen and water vapour can also be very important for wound dressings. A very high water permeability might lead to wound dehydration, whereas limiting oxygen afflux can promote angiogenesis through hypoxia [213].

The diffusion rates of both water and oxygen have been found to decrease with increasing zinc oxide content. Priyadarshi and Negi measured the water-vapour-transmission rate (WVTR) of chitosan/nZnO films [48]. In their study, the measured WVTR of chitosan films was 0.0067 g/m^2^/day, which decreased to 0.0028 and 0.0011 g/m^2^/day upon the addition of nZnO in amounts equal to 1% and 2% *w*/*w*, respectively. The decrease was attributed to the occupation of pore volume by the nZnO. Arfat and co-workers studied the properties of fish protein isolate/fish skin gelatin–ZnO BNCs [81]. 

The researchers measured a concentration-dependent decrease in water vapour permeability (WVP) up to a 3% *w*/*w* nano-ZnO loading. At 4% loadings, agglomeration determined the loss of matrix integrity, thus causing an increase in WVP. In the gelatin/β-glucan/ZnO nanocomposites prepared by Azari et al. [85], the WVP decreased from 2.15 × 10^−8^ g m^−1^ h^−1^ Pa^−1^ for neat gelatin films to 1.58 × 10^−8^ g m^−1^ h^−1^ Pa^−1^ for composite films containing 20% *w*/*w* β-glucans and 5% *w*/*w* nZnO. The decrease was explained by an increase in matrix compactness induced by the higher number of intermolecular interactions, as well as with the higher tortuosity imposed on the path of water molecules. Peighambardoust et al. [65] found similar results for starch-based BNC films. Furthermore, the authors found that nZnO was more efficient in decreasing WVP compared with CuO and Ag NPs owing to its superior dispersibility, which ultimately translated into maximum path tortuosity. Chu and co-workers [204] measured, instead, an increase in WVP for a polylactic acid film upon the addition of nZnO. However, a possible explanation for that can be found in the microstructural changes imparted to the continuous polymeric phase by the nano-filler that introduced some voids to the matrix, as shown by the SEM images. A higher water vapour permeability was also observed in the study by Shankar et al. [56], and was attributed to the higher porosity of the composite in the proximity of the separation between the gelatin film and the filler phases.

As for oxygen permeability, Indumathi et al. [71] reported a concentration-dependent decrease in the oxygen transmission rate (OTR) in chitosan/cellulose acetate phthalate/nZnO films, with a minimum of 1490.43 cm^3^ m^−2^ d^−1^ in correspondence with the highest nZnO loading of 7.5% *w*/*w*. This value was lower than that of traditional synthetic polymeric films, such as polyethene. The same was observed by Petchwattana et al. in poly(butylene succinate)/nZnO-BNC films [203]. The presence of nZnO constitutes an obstacle to oxygen molecules’ migration across the material. Similar results have been observed in numerous other studies [62,66,68,71,80,158,190,192,207,214].

#### 4.2.5. Ultraviolet Light Barrier

Wound dressings shall also be capable of protecting the injured area from harmful radiation. As a semiconductor with a band energy gap of around 3.3 eV, ZnO has excellent UV-blocking properties. Quantum confinement effects at the nanoscale can also increase the energy gap, thereby promoting the absorption of even higher frequencies of the spectrum. This property is always successfully transferred to the nanocomposites [56].

## 5. Applications in Wound Healing

### 5.1. Controlled Drug Release

nZnO-BNCs can be a promising platform for the controlled delivery of substances and agents that stimulate the healing process. In fact, the ability to release drugs loaded into the matrix is also modified by the simultaneous presence of nZnO in the system, as shown by Rath [212] for cefazolin-loaded nanofibrous gelatin mats, with incorporated nZnO, for surgical wounds. When designing a drug-delivery device, a controlled release profile is highly desired, as well as an optimal drug-loading capacity. Researchers have found that the nZnO content is an active parameter in optimising the drug-loading capacity [51,196,215] due to its previously discussed ability to influence the water uptake and swelling behaviour of the BNCs. In addition to this, drug release is also found to become slower and more controlled in proportion to the amount of nZnO included in the matrix. This may be due to the higher tortuosity that the nanofiller imposes on the path of the drug molecules upon release [197].

### 5.2. Antimicrobial Properties

As mentioned in Section 3.2, nZnO acts through three main antimicrobial mechanisms. These shall be reconsidered in the context of the BNCs and their application as a wound dressing.

The matrix can influence the release kinetics of zinc ions or of the nZnO itself, thus regulating the antimicrobial activity of the BNCs. Jebel et al. [53] and Villanueva and co-workers [79] examined the release properties of zinc ions or nZnO from bacterial cellulose (a polysaccharide) and keratin (a protein) BNC film and hydrogel, respectively. In the first case, it was found that greater release occurred in a medium with pH 8 as opposed to a medium with pH 4. This was attributed to the higher SR achieved under basic conditions, which promoted the diffusion of ions and nZnO. In the second case, it was found that two-month-old samples showed significantly lower release, presumably due to a slow rearrangement of molecules occurring during ageing and that translated into greater hindrance to diffusion. Therefore, both the pH and the ageing phenomena of the matrix were shown to play a role. This is relevant not only for the final application, but also for the storage conditions and time prior to use.

Release phenomena—of zinc ions or of nZnO particles—have also been investigated for other BNCs. Sudheesh Kumar [73] showed that 12% of the nZnO contained inside a β-chitin hydrogel was released after 1 week of immersion in phosphate-buffered saline. Azizi and co-workers investigated the release of Zn^2+^ from a poly(vinyl alcohol)-chitosan BNC film [52] through the inductively coupled plasma technique in water and showed a sustained and nZnO-concentration-dependent release of zinc ions in water throughout a period of 8 days, reaching a final concentration of 0.7 mg/L. In a work by Chu [204], the antimicrobial activity of a polylactic acid/nZnO film against *E. coli* was measured immediately after exposure to the samples and after 12 h. Inhibition was only reported after 12 h and attributed to the gradual release of nZnO from the porous matrix.

In general, the antimicrobial efficacy of nZnO-BNCs increases with the content of nanofiller [216,217], although in some cases, agglomeration might cause a reduction in the antimicrobial potential of nZnO [100]. In some systems, such as chitosan-based BNCs, the matrix itself possesses antimicrobial properties that can act in synergy with nZnO [48]. Carefully tuning the amounts of nZnO loaded in the composite films is important to maximise the antibacterial action without inducing cytotoxicity side effects to surrounding dermal cells [33]. As will be shown in the next section, nZnO-BNCs have actually been shown to be safe in several in vivo studies.

**Figure 5:**
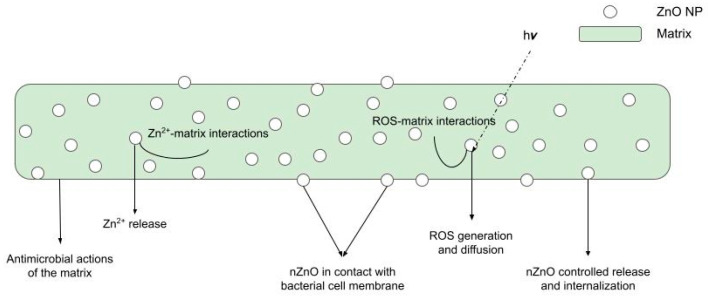
Possible antibacterial action mechanisms of BNCs.

Figure 5 below shows a schematic representation of all these phenomena at work.

Regarding nZnO, most BNCs tend to have greater inhibitory activity against Gram-positive bacteria, while a minority are more effective against Gram-negative bacteria; main results are summarized in Table 4. In particular, around 65% of the studies considered for the present review, employing the disk-diffusion method, reported a more marked effect on Gram-positive species. Examples of these nZnO-BNCs include carboxymethyl–chitosan/ZnO [58], oxidised starch/ZnO [64], cotton–starch/ZnO [63], keratin [79], fish protein isolate–fish skin gelatin/ZnO [80], bacterial cellulose [50], and many other BNCs obtained from either proteins or polysaccharides [47,48,85,194,200,207,210,218,219,220]. Similar to the case of bare nZnO, also in this case, the lower effect against Gram-negative bacteria is attributed to the lipopolysaccharide outer membrane covering their thin peptidoglycan layer, which would protect them from the antimicrobial species released by the nano-oxide.

On the other hand, nZnO-BNCs that are more effective against Gram-negative species than Gram-positive species include hyaluronic acid/nZnO [75] and chitosan/ZnO BNCs [83,100,166,189]. The reported results are attributed to the fact that the thicker peptidoglycan layer of Gram-positive bacteria is more difficult to penetrate by antimicrobial agents.

However, no sufficient evidence has been found to correlate the differences in the experimental conditions or in antimicrobial systems with these asymmetric results. One of the main reasons might be found in the use of different bacterial species and strains in these studies, which could have different susceptibilities to antimicrobials. Ahmed et al. [101] tested the antimicrobial efficacy of a chitosan–poly(vinyl alcohol) nanofibrous blend against *E. coli* and *S. aureus*, both in the presence and in the absence of nZnO. The results of the disk-diffusion method for the composite blend showed comparable inhibition zones of 20.2 ± 1 mm and 21.5 ± 0.5 mm, respectively, while the inhibition zones of the neat chitosan-poly(vinyl alcohol) blend were 14 ± 0.5 mm and 5 ± 0.5 mm, suggesting that the matrix may play a role in complementing the weaker effect of nZnO against Gram-negative bacteria. This is supported by several studies on the ability of chitosan to disrupt the Gram-negative cell membrane by means of electrostatic interactions [221,222,223]. Chitosan matrixes are indeed the most frequent among the abovementioned studies reporting greater activity against Gram-negative bacteria [83,100,166,189]. Similar results can be found in [200], which shows how chitosan-only films have greater antimicrobial activity against *E. coli* than against *S. aureus*. Data in [71] showed instead that chitosan-only films were more effective against *S. aureus* than against *E. coli* in an analogous disk-diffusion test, even though the chitosan used for film preparation had the same molecular weight in both studies and comparable deacetylation degrees. However, neither of the two studies reported the *S. aureus* and *E. coli* strains used, which might have explained the different results.

To elucidate the role played by the matrix, Kanmani and Rhim [68] prepared three different BNCs via an in situ method combining the same type of nZnO with three matrixes, namely agar, carrageenan, and carboxymethylcellulose. The matrixes altered the final aggregation and dispersion of the nanofiller. Additionally, the agar/nZnO composite was found to be less transparent to ROS-inducing UV light. The carrageenan/nZnO composite exhibited the lowest moisture content and water vapour permeability. The differences in these three matrixes did not result in any significant relative differences in the antimicrobial activity of the nanocomposites against the pathogens *E. coli* O157:H7 ATCC 43895 and *L. monocytogenes* ATCC 1531. However, Gram-positive *L. monocytogenes* was always found to be more susceptible to the BNC than *E. coli.*

On the other hand, to evaluate the role of morphologically different ZnO nanostructures coupled with the same matrix, Shankar et al. [56] tested nZnO obtained from different precursors in the same gelatin matrix against *E. coli* (Gram-negative) and *L. monocytogenes* (Gram-positive). Additionally, in this case, no significant difference in antimicrobial efficacy was observed, even though it could be noticed that the nZnO obtained from zinc acetate was slightly more effective against *L. monocytogenes* than the nZnO obtained from zinc nitrate.

These studies confirm that BNCs are effective against a wide variety of bacterial strains. This is fundamental to prevent wound infection and subsequent delayed or impaired healing, with potentially fatal consequences for patients. Still, many questions remain open about their actual mechanism of action. Moreover, it shall be noted here that most of the antimicrobial studies conducted did not test the BNCs against biofilms, which are the most common form in which bacteria are found in wounds [224]. This stands as another important gap that research should address to make a significant leap forward. Gaining insights into these aspects will, therefore, make a great contribution to a more rational design of better solutions. Additionally, research is also advancing to answer another important question: are BNCs effective in healing wounds?

**Table 4:** Antimicrobial efficacy of different nZnO-BNCs against different strains measured either via disk-diffusion tests or end-point assays.

Matrix Type	nZnO (% wt)	Test Type *	Tested Microorganisms	Test Results **	Ref.
Polyurethane/chitosan with mahua oil	5	Disk diffusion (10)	*S. aureus*	20	[99]
			*E. coli*	25	
Chitosan/Poly(vinyl alcohol)	5	Disk diffusion	*S. aureus*	14	[100]
			*E. coli*	17	
			*C. albicans*	2	
			*A. niger*	2	
Gelatin/hyaluronic acid/chitosan and asiatic acid	-	Disk diffusion (3)	*S. aureus*	4.9 ± 0.6	[83]
			*E. coli*	5.3 ± 0.2	
Alginate	5	Disk diffusion (13)	*MRSA*	15 ± 2	[41]
Chitosan	2	End-point assay	*B. subtilis*	3 mg/mL	[48]
			*E. coli*	6 mg/mL	
Bacterial cellulose	-	Disk diffusion (20)	*E. coli*	27 ± 0	[50]
			*P. aeruginosa*	25 ± 1	
			*S. aureus*	28.6 ± 1.15	
			*C. freundii*	26 ± 0	
Chitosan–cellulose	10–30 (% *w*/*v*)	Disk diffusion	*S. aureus*	13.75 ± 1.50	[51]
			*T. rubrum*	12.00 ± 1.82	
PVA/chitosan/CNC	5	Disk diffusion	*S. aureus*	6.3	[52]
			*S. choleraesuis*	4.9	
Bacterial cellulose	5	Disk diffusion (5)	*S. aureus*	11.8	[53]
			*E. coli*	5.6	
Carboxymethylcellulose	-	Disk diffusion (10)	*S. aureus*	13	[54]
			*B. subtilis*	16	
			*P. aeruginosa*	20	
			*E. coli*	16	
Gelatin	-	End-point assay	*L. monocytogenes*	1 LogCFU/mL after 12 h	[56]
			*E. coli*	c.a. 5.3 LogCFU/mL after 12 h	
Carboxymethylchitosan	-	End-point assay	*S. aureus*	99% reduction in viability after 4 h	[58]
			*E. coli*	99% reduction in viability after 6 h	
Chitosan–carboxymethyl cellulose–oleic acid	2	Disk diffusion (4)	*A. niger*	30.10 ± 1.50	[59]
Gelatin-chitosan nanofibers	5	Disk diffusion (12)	*E. coli*	25.06 ± 0.24	[84]
			*S. aureus*	33.13 ± 0.67	
			*P. aeruginosa*	12.95 ± 0.18	
Gelatin/β-glucan	-	Disk diffusion (12)	*S. typhimurium*	13.2 ± 1.72	[85]
			*E. coli*	15.0 ± 1.52	
			*S. aureus*	17.0 ± 2.10	
			*P. aeruginosa*	14.2 ± 1.32	
Gum acacia/poly(acrylate)	-	Disk diffusion	*E. coli*	32 ± 0.7	[61]
Hyaluronic acid	-	Disk diffusion (6)	*E. coli*	19	[75]
			*S. aureus*	11	
Methacrylated hyaluronic acid/elastin-like polypeptide	0.2	End-point assay	*MRSA*	28.3 ± 4.7 CFU	[76]
Keratin	5	Disk diffusion (5)	*E. coli*	7.7 ± 1	[79]
			*S. aureus*	13.5 ± 1.3	
Sago-starch	5	Disk diffusion	*S. aureus*	80 mm^2^	[62]
Oxidised starch	-	Disk diffusion (5)	*S. aureus*	100% inhibition	[64]
			*E. coli*	8–11 mm	
Carrageenan	-	End-point assay	*L. monocytogenes*	9 LogCFU/mL after 12 h	[66]
			*E. coli*	0 LogCFU/mL after 12 h	
Chitosan/cellulose/acetate phthalate	5	Disk diffusion (10)	*S. aureus*	23 ± 0.35	[71]
			*E. coli*	24 ± 0. 47	
Poly(lactic acid)	-	End-point assay	*E. coli*	3.31 LogCFU/mL after 12 h	[204]
Poly(vinyl alcohol)/chitosan	1	Disk diffusion (20)	*S. aureus*	26	[192]
			*E. coli*	25	
Poly(caprolactone) nanofibers	5	Disk diffusion (6)	*E. coli*	8.76 ± 1.2	[194]
			*S. aureus*	9.98 ± 0.6	
Chitosan	-	End-point assay	*E. coli*	2.5 ± 0.421 × 10^7^ CFU/g	[189]
			*S. aureus*	9 ± 0.367 × 10^7^	
Regenerated bacterial cellulose	2	Disk diffusion (15)	*E. coli*	41	[191]
Cellulose	-	Disk diffusion	*S. aureus*	10.40 ± 0.50	[196]
			*T. rubrum*	9.20 ± 0.20	
Starch	5	End-point assay	*E. coli*	~65% inhibitory rate	[207]
			*S. aureus*	100% inhibitory rate	
Chitosan–cellulose	-	Disk diffusion	*S. aureus*	13.75 ± 1.50	[51]
			*T. rubrum*	12.00 ± 1.82	
Gelatin nanofibers	3	Disk diffusion (10)	*S. aureus*	27 ± 1	[212]
Hydroxyethylcellulose	0.2	End-point assay	*E. coli*	60.2% inhibition	[225]
			*S. aureus*	91.5% inhibition	
Poly(lactic acid)/acetylated cellulose nanocrystals	5	End-point assay	*S. aureus*	100% growth inhibition rate	[226]
			*E. coli*	100% growth inhibition rate	
Carboxymethylcellulose	-	Disk diffusion (5)	*E. coli*	20 ± 2	[227]
			*S. aureus*	28 ± 2	
Chitosan/poly(vinyl alcohol) nanofiber	-	Disk diffusion (15)	*E. coli*	20.2 ± 1.0	[101]
			*B. subtilis*	15.5 ± 0.8	
			*S. aureus*	21.5 ± 0.5	
			*P. aeruginosa*	21.8 ± 1.5	
Cellulose/chitosan nanofibers	5	Disk diffusion (12)	*E. coli*	25.06 ± 0.24	[228]
			*S. aureus*	33.13 ± 0.67	
			*P. aeruginosa*	12.95 ± 0.18	
Ultrasonicated poly(lactic acid)	1	Disk diffusion (10)	*E. coli*	21.17 ± 0.07	[229]
			*S. aureus*	18.13 ± 0.08	
Bacterial cellulose	-	Disk diffusion (6)	*E. coli*	0	[230]
			*B. subtilis*	4 ± 0.13	
			*C. albicans*	0	

* For disk-diffusion tests, the number between parentheses indicates the diameter of the disk reported in the reference study, expressed in millimetres. ** For disk-diffusion tests, the number indicates the diameter of the inhibition zone, expressed in millimetres unless otherwise specified.

### 5.3. Wound-Healing Properties

Zinc oxide and biomacromolecules have been combined in different ways to promote wound healing. Blinov and co-workers formulated different gels by combining nano- and micro-ZnO with several biomacromolecules, namely maltodextrin, agar–agar, methylcellulose, amylopectin, and hydroxyethylcellulose [231]. The latter was found to be optimal for gel preparation and was used to treat burns on mongrel white rats, either alone or in combination with micro- or nano-zinc oxide. The researchers found that adding micro-ZnO to a hydroxyethylcellulose gel could boost the healing rate of burns in rats by 16.23% compared with the sole polysaccharide gel. These improvements increased to 24.33% when micro-ZnO was replaced with nZnO, confirming the promising properties of this oxide in its nanostructured form.

Biomacromolecules and nZnO have also been used to functionalise cotton pads, as reported in Hasanin [232]. Cotton pads functionalised with chitosan, glycogen, and nZnO led to a 99.73 ± 0.24% reduction in wound area in Wistar rats after 17 days, compared with an 89.14 ± 0.97% reduction in the control group treated only with a cotton pad.

In 2013, Kumar et al. [73] tested the wound-healing potential of a β-chitin hydrogel/nZnO composite bandage as a potential solution for different types of infected wounds with large volumes of exudate. Bandages were tested on Sprague–Dawley rats, facilitating a 100% skin wound closure after 3 weeks, together with good antimicrobial activity and collagen deposition. 

Rakhshaei and Namazi [233] prepared a nanocomposite hydrogel based on carboxymethyl cellulose filled with nZnO-impregnated MCM-41 mesoporous silica, loaded with tetracycline, a broad-spectrum antibiotic. In addition to the enhancement of the antimicrobial properties against *E. coli* brought by nZnO, a cytotoxicity test was carried out using adipose tissue-derived stem cells. The tests showed 63% cell viability after 24 h of the nZnO-containing hydrogels, compared with 81% in nZnO-free hydrogels. However, the viability continued increasing for the next 7 days in both cases. Similar results were obtained by Khorasani et al. [192] for a heparinised nZnO/poly(vinyl alcohol)/carboxymethyl cellulose hydrogel tested on mouse fibroblast cells (L-929). These results led to the conclusion that the BNCs were, in fact, biocompatible.

Along the same line, Raguvaran et al. [234] demonstrated that embedding nZnO into sodium alginate–gum acacia hydrogels yielded remarkably lower cytotoxicity than the bare nano-oxide while retaining good wound-healing properties. In fact, the hydrogel matrix was able to effectively reduce the contact between nZnO and the cells, and to control Zn^2+^ release. This is reassuring evidence, considering the existing concerns about the toxicity of nZnO.

Burn wounds on Bagg’s albino (BALB) mice were treated with bacterial cellulose/nZnO composites by Khalid et al. [50], who reported wound-healing activity comparable to that of a commercial silver sulfadiazine cream. Histopathological analyses revealed regenerated epithelium in the wound beds treated with the BNC. After 15 days, the average wound area was 98.3 ± 7.6 mm^2^ after treatment with the BNCs, compared with 143 ± 7.5 mm^2^ for the bacterial cellulose dressing alone and 66 ± 5.7 mm^2^ for the positive control.

In a similar study, re-epithelialisation, dense keratinocytes proliferation, and organised fibrous connective tissue were observed by Lu and coworkers [44] after wound treatment with a chitosan/nZnO composite. The composite performed better than a commercial ZnO ointment gauze. Promising results were also obtained by Amhed and co-workers [101] on diabetic rabbits treated with an electrospun chitosan/poly(vinyl alcohol) nanofiber mat loaded with nZnO. The BNC induced a 44.8 ± 4.9% wound contraction by day 4, whereas the ZnO-free mat only led to a 22.5 ± 3.0% contraction.

## 6. Conclusions

In the present review, the overview of findings clearly shows the promising role of BNCs containing nZnO in wound-healing applications. It provides an extensive examination of nZnO-BNCs’ preparations and applications, showing how biomacromolecules and nZnO interact based on their type and on the preparation processes employed, and how such factors determine their final properties. At the same time, this work highlights the main challenges to be addressed in order to develop these materials to an advanced stage. Among the main concerns, the comprehension of the antibacterial mechanism, influenced by different variables, has to be carefully investigated. To this end, the chosen antimicrobial test protocol, as well as the bacterial strains tested, are fundamental. For instance, the presence of an outer membrane in Gram-negative bacteria is likely not sufficient to provide satisfactory answers to the different results reported in antibacterial tests.

Gaining a deeper understanding of all these aspects through an extensive review is essential to enable the scientific community to cooperate and work towards better and more effective wound therapies. A fundamental aspect is the development of new and affordable medical devices that are able to improve and accelerate the wound-healing process, particularly in patients affected by burns or chronic wounds. In fact, among the skin wound types, burns or chronic wounds imply long hospitalization because they are often worsened by antimicrobial-resistant pathogens. These kinds of wounds have to be treated with dressings characterised by good water retention, gas permeability, flexibility, and antibacterial action, such as nZnO-BNCs dressings. The vast range of biomacromolecules and types of nanostructured zinc oxides, the ease of fabrication, and the possibility of tuning properties, such as the swelling ratio, stiffness and elongation, permeability, and antimicrobial action, offers unprecedented opportunities to craft targeted solutions and to deliver significant progress to wound care.

## Figures and Tables

**Table 1:** Overview of biomacromolecules for wound dressing films.

Biopolymer	Properties	Refs.
Polysaccharides		
Alginate	Biocompatible, non-toxic, non-immunogenic, biodegradable, antimicrobial, and haemostatic	[39,41,42,43]
Chitosan	Non-toxic, biocompatible, biodegradable, moisture retentive, and haemostatic	[44,45,46,47,48]
Chitosan oligosaccharide	Antibacterial, anti-inflammatory, and immune-stimulating	[49]
Cellulose	Biocompatible, hydrophilic, microporous, transparent, and non-toxic	[50,51,52,53]
Carboxymethyl-cellulose	Biocompatible and biodegradable	[54,55,56,57]
Carboxymethyl-chitosan	Amphoteric and hydrophilic	[58,59,60]
Gum acacia	Biocompatible, nontoxic, and water-soluble	[61]
Starch	Low-cost, biocompatible, biodegradable, easy preparation, and good film-forming properties	[62,63,64,65]
Carrageenan	Water-soluble, and good film-forming abilities and mechanical properties	[66,67,68]
Cellulose acetate	Water-insoluble, high transparency, and good mechanical and chemical resistance	[69,70]
Cellulose acetate phthalate	Hydrophilic and biodegradable	[71]
Oxidised starch	Low viscosity, and high stability and transparency	[64]
Dextran	Active in wound healing and controls bacterial growth	[39,72]
Chitin	Biodegradable, haemostatic, and cytocompatible	[73]
β-glucans	Immunostimulatory, and biodegradable	[74]
Hyaluronic Acid	Primary component of connective tissue, safe long-term, and reduces bacterial adhesion	[75,76]
Proteins		
Collagen	High biocompatibility and biodegradability. Bioactive	[77]
Keratin	Low-cost and highly suitable for hydrogels, but concerns about wastes	[78,79]
Fish protein isolate	High film-forming ability	[80,81,82]
Gelatin	Biodegradable, biocompatible, and cell-recognizable	[39,56,81,83,84,85]
Soy protein isolate	Biocompatible, non-immunogenic, non-toxic, low-cost, biodegradable, and highly stable	[86]
Silk fibroin	Biocompatible, biodegradable, and minimum inflammatory reactions	[87]

**Table 2:** Effect of nZnO on the elastic modulus (EM) and tensile strength (TS) of films.

Matrix Type	EM (mPa)		TS (Mpa)		nZnO (% wt)	Refs.
	Pure	Filled	Pure	Filled		
Agar	1004.9	109.8	34.6	13.0	-	[68]
Carrageenan	1112.1	130.4	44.6	12.3	-	[68]
Carboxymethyl-cellulose	20.5	14.0	6.4	5.1	-	[68]
Gelatin	1451.2	262.5–344.4	50.1	29.8–33.4	-	[56]
Chitosan	1821	3304	12.84	41.73	1–2	[48,189]
Starch	7.8	25.44	4.11	12.73	2–3	[65,207]
Poly(lactic acid)/poly(butyleneadipate-co-terephthalate	800	970–1220	14.5	16.8–26.9	3	[190]
Bacterial cellulose	93.8	132.11	26.3	45.21	5	[53]
Poly(lactic acid)	3118.8	2610.64	47.78	39.96	3	[204]
Poly(caprolactone) nanofibers	3.70	5.25–3.78	1.40	1.60–0.98	1–6	[194]

**Table 3:** Effect of nZnO’s presence on the WCA of different films.

Matrix Type	WCA (deg)		nZnO (% wt)	Refs.
	Pure	Filled		
Agar	66.5	68.8	-	[68]
Carrageenan	61.6	84.5	-	[68]
Carboxymethyl-cellulose	31.6	55.2	-	[68]
Gelatin	52.4	62.15	-	[56]
Starch	51	43	2–3	[65,207]
Poly(lactic acid)/poly(butyleneadipate-co-terephthalate	82	78.1–88.3	3	[190]
Chitosan-cellulose acetate phthalate	57	81	5	[71]
Cellulose acetate nanofibers	47	124	-	[210]
